# Physiological and transcriptomic analysis provide novel insight into cobalt stress responses in willow

**DOI:** 10.1038/s41598-020-59177-y

**Published:** 2020-02-11

**Authors:** Yi-Ming Wang, Qi Yang, Hui Xu, Yan-Jing Liu, Hai-Ling Yang

**Affiliations:** 1 College of Biological Sciences and Biotechnology, Beijing Forestry University, Beijing, 100083 China; 2 Umeå Plant Science Centre, Department of Plant Physiology, Umeå University, SE 901 87 Umeå, Sweden; 3 State Key Laboratory of Tree Genetics and Breeding, Chinese Academy of Forestry, Beijing, 100091 China

**Keywords:** Transcriptomics, Abiotic

## Abstract

Cobalt (Co) is an essential component of several enzymes and coenzymes in living organisms. Excess Co is highly toxic to plants. The knowledge of molecular response mechanisms to cobalt stress in plants is still limited, especially in woody plants. The responses of weeping willow (*Salix babylonica*) seedlings to Co stress were studied using morphological and physiochemical measurements and RNA-seq analysis. The physiological and biochemical indexes such as growth rate, the content of chlorophyll and soluble sugar, photosynthesis and peroxidase activity were all changed in willow seedlings under Co stress. The metal ion concentrations in willow including Cu, Zn and Mg were disturbed due to excess Co. Of 2002 differentially expressed genes (DEGs), 1165 were root-specific DEGs and 837 were stem and leaf-specific DEGs. Further analysis of DEGs showed there were multiple complex cascades in the response network at the transcriptome level under Co stress. Detailed elucidation of responses to oxidative stress, phytohormone signaling-related genes and transcription factors (TFs), and detoxification of excess cellular Co ion indicated the various defense mechanisms in plants respond to cobalt stress. Our findings provide new and comprehensive insights into the plant tolerance to excess Co stress.

## Introduction

Heavy metal elements present in the soil can be taken up into plant tissues along with nutrients. Heavy metals, such as cadmium (Cd), copper (Cu), cobalt (Co), manganese (Mn) and nickel (Ni), are toxic to plants when absorbed in excessive amounts^1^. Environmental pollution involving toxic metals has accelerated which become a major challenge for plants and other organisms nowadays. Cobalt is a transition metal and is used in hard metal industry. Its pollution mainly originates through wastewater from industry, manufacture, processing, use and disposal of cobalt-containing products^2^.

Cobalt is one of the beneficial elements that promote plant growth and may be essential to particular taxa but not for all plant species^3^. Low levels of Co concentration had beneficial effects on growth, seedpod yield and nodule development in leguminous plants, such as faba bean (*Vicia faba*) and soybean^4,5^. It is an integral component of cobalamin (vitamin B12), which is required for the activity of several enzymes involved in nitrogen-fixation^6^. The beneficial effects are associated with symbiotic rhizobia that inhabit nodules of legumes^6^. In addition, Co was reported to play a role in delay of leaf senescence through inhibition of ethylene biosynthesis and increasing drought resistance^3^. However, excess Co was toxic to higher plants as showed by studied in various species, such as barley^7^, oilseed rape^8^, tomato^9^, cauliflower^10^ and mung bean^11^. Co toxicity could cause irreversible damages to plants via oxidative stress, iron deficiency and photosynthesis inhibition. The study in mung bean showed Co could disrupt iron homeostasis probably by competing with iron for access to transporters^11^. Excess of Co interfered with the enzymes involved in chlorophyll biosynthesis, causing a decrease in chlorophyll concentration and affecting protein content and various antioxidants activities^12^.

Most studies on cobalt stress in plants focused on the physiological responses and/or functions of individual genes. Previous study showed IRON REGULATED TRANSPORTER 1 (IRT1) protein, an *Arabidopsis thaliana* metal ion transporter, transported Co to the root epidermal cells^13^. IRT1 expression was induced by heterodimers of the FER-like IRON-DEFICIENCY INDUCED TRANSCRIPTION FACTOR (FIT) with the basic helix-loop-helix (bHLH) transcription factor (bHLH38 or bHLH39)^14^. In addition, heavy metal associated 3 (HMA3) protein could transport the excess cobalt ions to vacuoles^15^.

Despite the numerous studies on physiological responses and functions of a few genes under Co stress, little is known about transcriptome changes and the mechanisms regulating gene expression in response to Co stress, especially in woody plants. In this study, we used weeping willow (*Salix babylonica*) to investigate the effects of excess cobalt. Weeping willow originates from China and is one of the common species in genus *Salix*. It is a tall deciduous tree with wide distribution due to its high viability and vitality, well-developed roots and rapid growth^16^. By comparing the physiological and biochemical indexes of the weeping willow growing under normal and Co stress conditions, we studied the physiological responses and explored the molecular mechanisms via RNA-seq. Our results provide novel insights into the Co responses in woody plants.

## Results and Discussion

**Figure 1:**
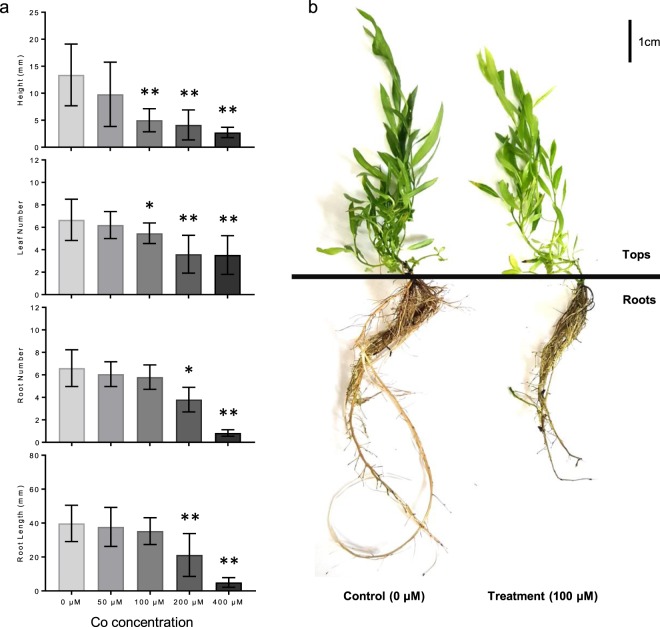
Effects on growth of willow tissue-cultured seedlings under Cobalt stress. (**a**) Phenotypic analyses of willow seedlings in response to different Cobalt concentrations treatments including seedling height, leaf number, root length and root number. Data represent mean ± SD (n = 15). Asterisks indicate the significant difference between control (0 μM) and Co treatments (*P ≤ 0.05; **P ≤ 0.01, t-test). The photo of control (0 μM) and Co treated (100 μM) seedlings are presented in (**b**). Seedlings were separated into two parts, Tops (including stem and leaf tissues) and Roots, as indicated with the black line for further analysis.

To study the effects of different Co concentrations on tree growth, stem segments of weeping willow (*S. babylonica*) were cultured on the woody plant medium (WPM) containing different concentration of Co^2+^ (0 μM, 50 μM, 100 μM, 200 μM and 400 μM) for 30 days. Tissue-cultured seedlings showed a stress phenotype with decreased height and reduced leaf number when the concentration of Co^2+^ was 100 μM (Fig. 1a). The length and number of roots were significantly decreased when the concentration of Co^2+^ was 200 μM or higher (Fig. 1a). Seedlings died gradually when cultured on the medium with 200 or 400 μM Co^2+^. Similar to the results against Co stress in chickpea^17^, the results presented in this study indicated that the lower and upper limit of tolerance to Co^2+^stress for willow seedlings were, respectively, 50 μM and 200 μM in the medium. Considering there was no significant effect on willow growth at low Co concentration (50 μM) and that high Co conditions (200 μM) was lethal, we selected the plants grown under moderate stress conditions of 100 μM Co^2+^ for further biochemical, physiological and transcriptomic analyses.

### *De novo* assembly and functional annotations of transcriptome sequences

To investigate the gene expression in response to cobalt stress in willow, seedlings grown at 0 μM Co (control) and 100 μM Co were separated into tops (stem and leaf tissues) and roots for the transcriptome sequencing (RNA-seq) analysis. A total of 741,005,204 raw reads were generated from 12 libraries and 713,205,946 high-quality clean reads were retained for subsequent analysis after quality control (Table S1). Transcriptome sequences were assembled *de novo* using the Trinity software, and 155,851 transcripts and 111,225 unigenes with N50 values of 1,321 and 1,240 bp, respectively, were obtained (Table S2). The results showed that 48.75% of transcripts and 41.92% of unigenes had a length of more than 500 bp. The quality of assembled unigenes was subsequently evaluated with BUSCO software. Approximately 87% of 1440 orthologous genes shared with embryophyta were assembled as complete genes (Table S3).

Each unigene was aligned to eight databases (NR, NT, Swiss-Prot, Pfam, KOG, KEGG, GO and Plant Transcription Factor Database) with E value < 10^−5^ for annotation. There were 59,080 unigenes that had annotation in at least one database. A total of 39,861 unigenes were annotated as homologs against the NR (non-redundant protein) database (Table S4). The species that provided the best BLAST hits was *Populus trichocarpa*, which showed homology to 47.27% unigenes of weeping willow (Fig. S1). There were 26,599 putative protein-coding genes identified in the whole genome of *Salix suchowensis* and 20,261 of which were homolgous genes with the *Populus trichocarpa* reference gene set^18^. The annotated unigenes in weeping willow (*S. babylonica*) were more than genes identified in *S. suchowensis* genome indicating the high quality of sequencing and assembly of our data. There were 27,670 unigenes annotated into 41 functional groups of three GO categories (biological process, cellular component and molecular function). There were more than 12,000 genes annotated into “cellular process” and “metabolic process” which ranked the highest in the biological process category (Fig. S2). The number of genes involved in “cell” and “cell part” groups were the most abundant in cellular component category (Fig. S2). In the molecular function category, “binding” and “catalytic activity” groups contained most genes corresponding to 14,115 and 12,825, respectively (Fig. S2). For annotation of transcription factors (TFs), 1,358 unigenes were identified as TFs (Table S5). These TFs were divided into 55 major categories, including bHLH (8.47%), ERF (8.17%), NAC (7%), MYB (6.55%) or C2H2 (5.89%) families (Fig. S3).

### Differential expression analysis and GO and KEGG enrichments under cobalt stress

**Figure 2:**
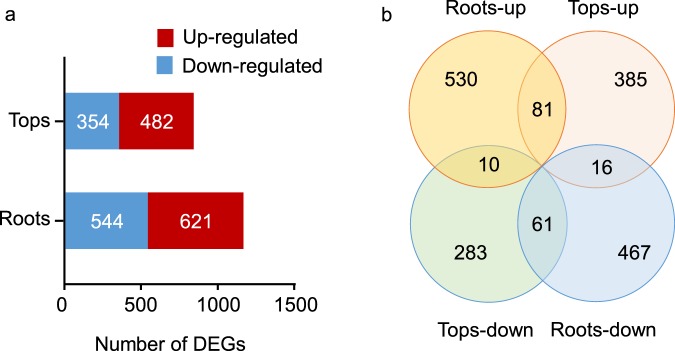
Identification of DEGs in willow under Co treatment (100 μM Co^2+^). (**a**) The total numbers of DEGs identified in Roots and Tops from willow seedlings of Co treated compare to control. DEGs that are up-regulated or down-regulated were represented by red and blue boxes, respectively. (**b**) Venn diagram comparing DEGs in Roots and Tops between Co treated and control seedlings. Indicated in the diagram are the numbers of up-regulated and down-regulated DEGs.

The gene expression levels were obtained through evaluation of read counts. The square of Pearson’s correlation coefficient (R^2^) was more than 0.94, indicating strong correlations among three independent replicates. Principal component analysis showed that the expression of three replicates was highly correlated and the control groups was clearly separated from the treatment groups (Fig. S4). Unigenes that matched the criteria of |log2 (Fold Change)| > 1 and FDR < 0.05 were identified as differentially expressed genes (DEGs). In total, 1165 and 837 DEGs were identified in roots and tops, respectively (Fig. 2a). A total of 168 DEGs were differentially expressed in both roots and tops. Among them, 81 DEGs were up-regulated and 61 DEGs were down-regulated in both tops and roots (Fig. 2b). There were 10 DEGs that were up-regulated in roots while down-regulated in tops (Fig. 2b). The remaining 16 DEGs were up-regulated in tops while down-regulated in roots (Fig. 2b). A total of 107 TFs were identified from all DEGs belonging to 23 TF families (Fig. S5 and Table S6). In roots, 64 TFs were identified as DEGs including 41 up-regulated and 23 down-regulated. There were 57 TFs in tops, with 31 up-regulated and 26 down-regulated (Fig. S5). The most abundant TFs belonged to NAC and ERF families (Fig. S5). To validate the accuracy and reproducibility of RNA-seq results, twelve DEGs were randomly chosen for qRT-PCR; these unigenes were up/down-regulated in our transcriptome data and encoded different proteins including transcription factors, transporter proteins, membrane proteins and oxidoreductases. The results of qPCR were consistent with the RNA-seq data, confirming the accuracy of the RNA-seq dataset (Fig. S6).

**Figure 3:**
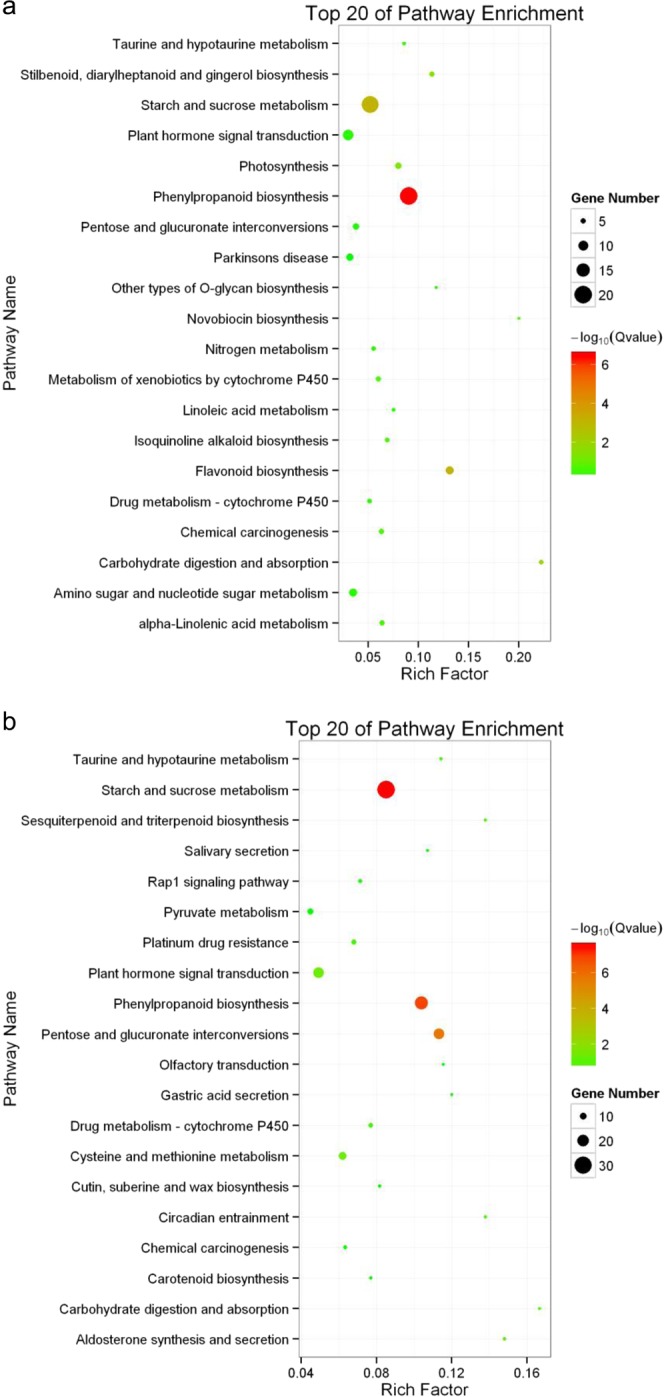
KEGG enrichment of DEGs in willow under Co treatment. Top 20 enriched pathways from Tops (**a**) and Roots (**b**) are presented, respectively. Rich factor indicated the ratio of enriched DEGs number to the total number of genes have been annotated in this pathway. The numbers of DEGs enriched in each pathway are indicated by circle size. The enrichment scores (−Log10 (Q value)) of each KEGG pathways are shown by color gradient.

In order to investigate the biological functions associated with the Co stress response in different organs, the GO enrichment analysis was performed for DEGs in roots and tops. There were about 300 over-represented GO categories corresponding to biological processes and molecular functions in tops under Co treatment (Table S7). Most DEGs from tops were enriched in processes such as “tetrapyrrole binding”, “heme binding”, “oxidation-reduction process”, “carbohydrate metabolic process”, “secondary metabolic process” and “iron ion binding”. While apart from these GO categories enriched in tops, the GO enrichments of DEGs in roots showed the “external encapsulating structure organization” and “cell wall organization” were also over-represented (Table S8). Apparently, these categories related to the response to abiotic stimuli and the iron-related processes. The KEGG enrichment of the DEGs in roots and tops were analyzed to identify pathways that involved in Co stress responses. Considering the top 20 most enriched KEGG pathways shown in Fig. 3, the DEGs of tops and roots were enriched in “Phenylpropanoid biosynthesis”, “Starch and sucrose metabolism”, “Flavonoid biosynthesis” and “Plant hormone signal transduction” pathways. And the DEGs of Roots were also enriched in “Cysteine and methionine Metabolism” pathways (Fig. 3b and Table S9). The combination of enrichment analyses indicated the antioxidant system and carbohydrate metabolism played important roles in response to Co stress.

### Hormonal signaling and transcriptional regulations under cobalt stress

**Figure 4:**
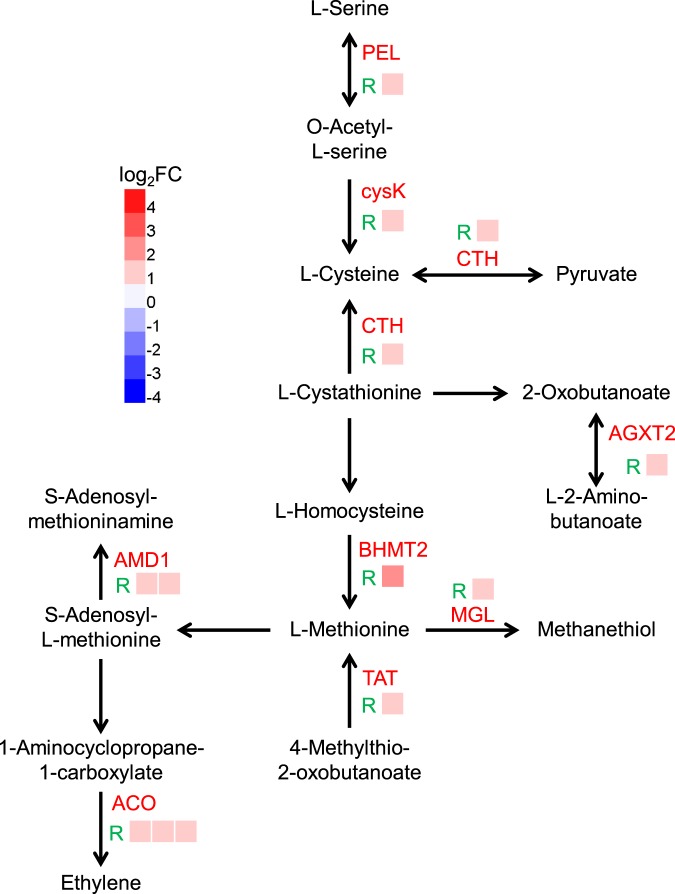
The differentially expressed genes enriched in cysteine and methionine metabolism pathway in willow in responding to Co stress. The names of DEGs encoded **e**nzymes involved in the pathway are indicated in red. The green capital letter R represents the DEGs were identified from roots. The expression profiles of each gene copy of DEGs are shown by colored squares. Expression levels are indicated by color gradient with red and blue representing up-regulation and down-regulation, respectively.

Phytohormones, such as abscisic acid (ABA), ethylene (ET), salicylic acid (SA), and jasmonic acid (JA), are crucial plant signaling molecules in responses to abiotic stress. A number of DEGs involved in different phytohormone signaling pathways were enriched under Co treatment (Fig. 3). Genes associated with ethylene biosynthesis pathway were substantially up-regulated by Co stress including genes involved in “Cysteine and methionine metabolism” (Fig. 4 and Table S9). Ethylene is involved in various molecular and physiological processes (e.g. senescence and defense responses), and it is one of the receptors induced by heavy metal stress^19,20^. A dozen of TFs belonging to the ethylene response factor (ERF) family were differentially expressed in willow under Co stress (Fig. S5). The previous study showed the expression of ethylene response DNA binding factor 4 (EDF4, AT1G13260) was induced when ethylene accelerated the leaf senescence^21^. The homolog of EDF4 in willow (c90798_g1) was up-regulated in the Co treatment (Table S6). In summary, these results suggested ethylene play an important role of in Co stress responses. However, the exact role of ethylene and the crosstalk between phytohormones to fine-tune the defense response to Co stress in willow remain to be discovered.

### Physiological responses and corelated transcriptome changes under cobalt stress

**Figure 5:**
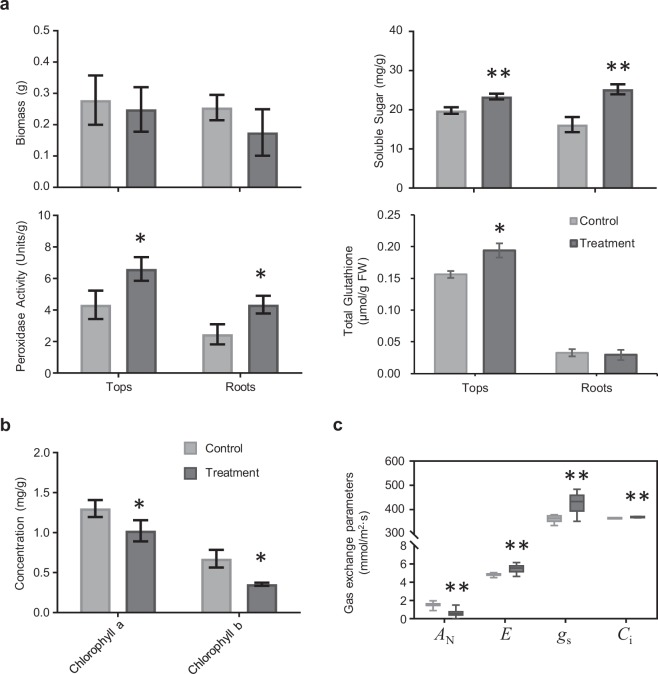
Various physiological analysis in willow seedlings under 100 μM Co^2+^ treatment. (**a**) Effect of cobalt stress on the biomass, soluble protein, peroxidase activity and total glutathione in different willow organs. (**b**) The concentration of chlorophylls in willow leaves. Data in (**a** and **b**) represent mean ± SD (n = 15). (**c**) Boxplots (means, boxes 25–75%, +/− bars 95%, n = 15) of photosynthesis parameters determined by gas exchange. *A*_N_, net photosynthetic rate (mmol/m^2^·s), *E*, transpiration rate (mmol/m^2^·s), *g*_s_, stomatal conductance (mmol/m^2^·s) and *C*_i_, intercellular carbon dioxide concentration (mmol/m^2^·s). Asterisks indicate the significant difference between treatment and control (*P ≤ 0.05; **P ≤ 0.01, t-test).

**Figure 6:**
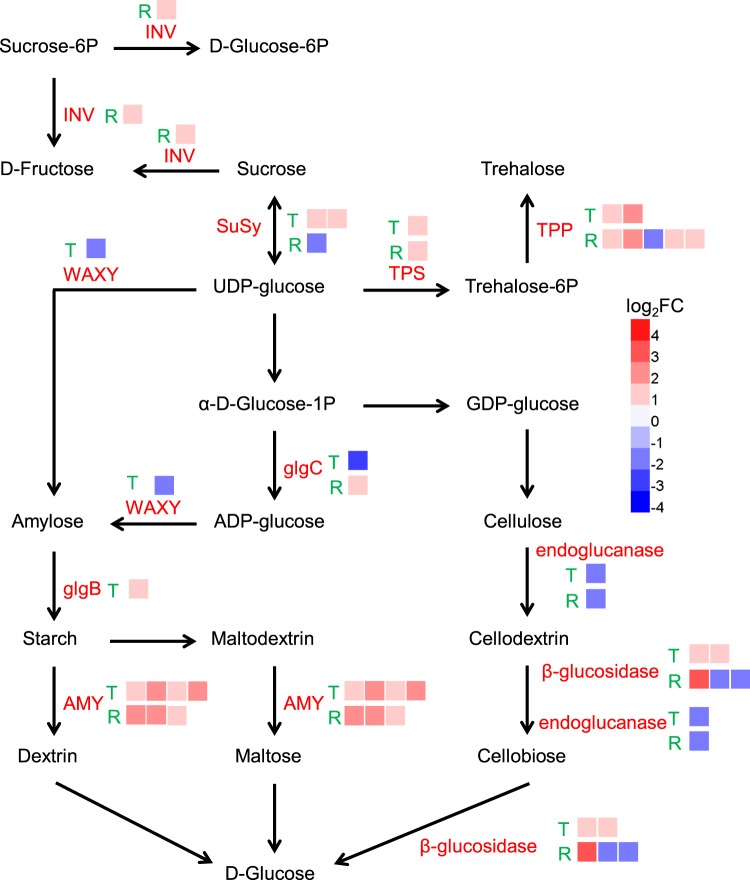
The differentially expressed genes enriched in starch and sucrose metabolism pathway in willow in responding to Co stress. The names of DEGs encoded **e**nzymes involved in the pathway are indicated in red. The green capital letters T and R represent the DEGs were identified from Tops (leaf and stem tissues) and roots, respectively. The expression profiles of each gene copy of DEGs are shown by colored squares. Expression levels are indicated by color gradient with red and blue representing up-regulation and down-regulation, respectively.

Cobalt has been shown to affect plant growth and metabolism^12,22^. In this study, the biomass of seedlings treated with Co was lower than in the control, although the difference was not significant (Figs. 1b and 5a). The content of chlorophyll *a* and *b* were both significantly reduced in Co treated leaves compared with control leaves (*P* < 0.05, Fig. 5b). The RNA-seq data demonstrated that the expression of genes involved in chlorophyll biosynthesis were inhibited under Co stress (Table S10). MYB116 (AT1G25340) in *A. thaliana* was involved in chlorophyll degradation^23^, and its homolog in willow (c84877_g2) was up-regulated under Co stress (Table S6). Clear signs of oxidative stress in the Co treatment presented in this study were similar to those reported for heavy metal stress in different plant species^1,10,12,19^. Excess Co is toxic by the generation of reactive oxygen species (ROS) that cause oxidative stress. The accumulation of soluble sugar was one of the common physiological responses to oxidative stress in plants. Soluble sugar content was increased under Co stress (Fig. 5a). Corresponding to the results on sugar content, a number of genes with functions in carbohydrate metabolic processes were identified in the DEGs enrichment analysis. Trehalose had been shown to protect plants from multiple abiotic stresses, it is synthesized mainly by the proteins encoded by trehalose-6-phosphate synthase genes (TPSs) and trehalose-6-phosphate phosphatase genes (TPPs)^24^. The pathway enrichment analysis found that the DEGs related to starch and sucrose metabolism pathway were over-represented including one TPS and four TPPs that were up-regulated (Figs. 3 and 6).

Plants have developed ROS detoxifying systems through antioxidants including enzymes and non-enzymatic antioxidant molecules. Peroxidase is one of the enzymes which are responsible for removal of H_2_O_2_^25^. The determination of peroxidase activity showed an increase in the Co treated seedlings (Fig. 5a). Consistent with increased peroxidase activity, 13 peroxidase genes were up-regulated in roots under Co stress (Table S9). In plant, methionine plays an important role in promoting the synthesis of glutathione (GSH), increasing the activity of glutathione peroxidase and superoxide dismutase, and maintaining the balance of ROS in plants under abiotic stress^26^. Many genes involved in “methionine metabolism” were up-regulated in roots under Co stress (Table S9 and Fig. 4). Accordingly, the total glutathione accumulated in tops under Co treatment (Fig. 5a). The previous studies have reported that the phenylpropanoid compounds play a critical role in the plant defense response as beneficial antioxidants^27^. Phenolic compounds (such as flavonoids and derivatives of hydroxycinnamic acids) were antioxidants defending against Cu stress in red cabbage^28^. DEGs were over-represented in “Phenylpropanoid biosynthesis” and “Flavonoid biosynthesis” pathways (Fig. 3 and Table S9). Some important enzymes of the phenylpropanoid pathway including cinnamate 4-hydroxylase (C4H, c89552_g1) and cinnamyl-alcohol dehydrogenase (CAD, c61680_g2) were also highly up-regulated.

### Disturbance of metal ions homeostasis and the detoxification response under cobalt stress

**Figure 7:**
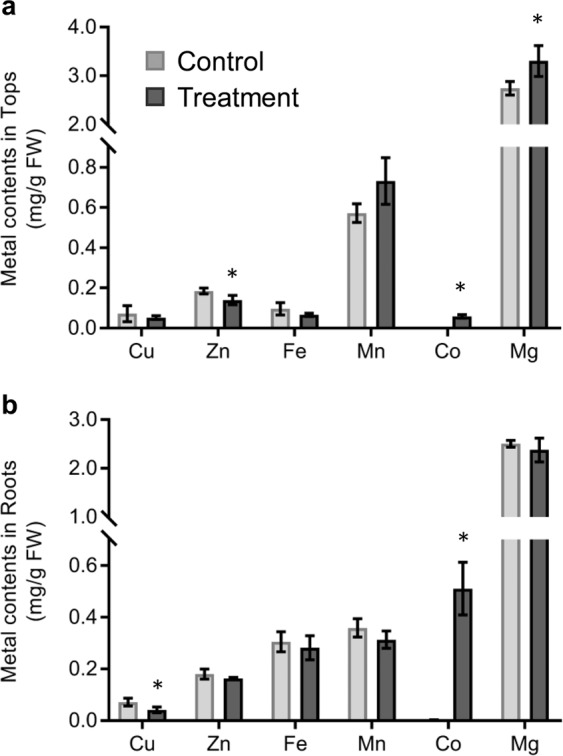
The contents of different metal elements in willow seedlings under 100 μM Co^2+^ treatment. (a) Metal contents in Tops (leaf and stem tissues). (**b**) Metal contents in Roots. Data represent mean ± SD (n = 3). Asterisks indicate the significant difference between treatment and control (*P ≤ 0.05; t-test).

Heavy metals such as Fe, Mn, Zn, Ni and Cu are essential nutrients for plants. Due to the low specificity of metal ion uptake and distribution systems in plants, the excess Co could be toxic by disturbing the homeostasis of other crucial metal ions. To determine the effect of excess cobalt on metal ion hemostasis in willow, we measured the contents of various metal ions. The Co concentration was increased more than 130-fold in both tops and roots under Co treatment (Fig. 7 and Table S11). The concentration of copper was decreased significantly only in roots under Co stress, whereas the zinc concentration was decreased significantly only in tops (independent *t*-test *P* < 0.05, Fig. 7). These results suggested the excess Co stress had different effects on uptake and transport of metal ions. In addition to the concentration changes of metal ions in plants under Co stress, another possible molecular mechanism of heavy metal toxicity could be the substitution of necessary metal ions in biomolecules^29^. Divalent cations (e.g. Co^2+^ and Ni^2+^) could displace Mg^2+^ in ribulose-1,5-bisphosphate-carboxylase/oxygenase (RuBisCO) which resulted in loss of activity^30^. The net CO_2_ assimilation rate (*A*_N_) decreased significantly in Co treated seedlings (Fig. 5c). However, the maximum photosynthesis efficiency (*F*_v_/*F*_m_) was not inhibited (Fig. S7) and intercellular CO2 concentration (*C*_i_) remained high in the Co treatment (Fig. 5c). These results suggested the inhibition of CO_2_ assimilation was probably due to the displacement of Mg^2+^ by Co^2+^ in RuBisCO resulting in an inactivated form. Interestingly, the leaf Mg concentration was increased in Co treated willow (Fig. 7a) potentially indicating a compensatory response for the reduced RuBisCO activity.

**Figure 8:**
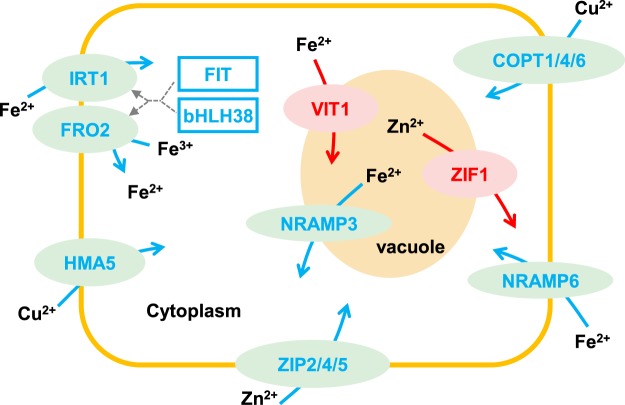
Differentially expressed genes related to metal ion uptake in willow roots under Co stress. Proteins encoded by down-regulated genes are shown in blue ellipses and up-regulated genes are shown in red ellipses. Down-regulated TF genes are indicated with blue boxes. Solid arrows show the directions of metal ion transportation or reduction. Dash arrows indicate the positive regulation of *FIT* and *bHLH38* to *IRT1* and *FRO2* genes.

Plant have developed complex defense mechanisms against heavy metal stress including down-regulating metal ion uptake rate, up-regulating excretion rate and increasing the metal binding molecules^1,31^. In the present study, the DEGs identified by transcriptomic analysis showed an enrichment in “iron ion binding” process in response to Co stress (Table S7). Most genes involved in metal ion homeostasis were down-regulated in willow under Co stress (Fig. 8 and Table S12). However, the Fe concentrations did not show significant changes under Co treatment. The study of transcriptional responses to iron deficiency in Arabidopsis suggested that plants induced a suite of transporters that compensated for a surplus of undesired metals to avoid imbalances in ion distribution^32^. IRT1 and FERRIC REDUCTION OXIDASE 2 (FRO2) play a key role in uptake of Fe^2+^ and other divalent metal cations, and their expression are regulated by FIT and bHLH38/bHLH39 TFs^14,33^. Transcriptomic data showed the expression of both *IRT1* and *FRO2* was down-regulated in Co-treated willow roots together with the upstream *FIT* and *bHLH38* as well (Fig. 8 and Table S12). Along with the suppression of iron uptake pathway, several metal transporter genes were also down-regulated under excess Co, such as *HEAVY METAL ATPASE 5* (*HMA5*) for Cu transport and *NRAMP METAL ION TRANSPORTER 6* (*NRAMP6*) for Fe transport (Fig. 8 and Table S12). The expression of *VACUOLAR IRON TRANSPORTER 1* (*VIT1*) that imports iron into the vacuole was up-regulated in roots (Fig. 8 and Table S12). The previous study showed that cysteine was involved in Co complexation in plants^34^. The Cys-rich protein family, phytochelatins, plays a critical role in metal detoxification via metal complexation^1^. GSH is a tripeptide that is the most abundant low molecular weight thiol in plants and the key component in sequestration of heavy metals. The thiol group provided by the cysteine in GSH is important in the formation of mercaptide bond with metals^1^. The total glutathione concentration was significantly induced in tops under Co stress (Fig. 5a). Corresponding to the accumulation of GSH, many genes related to cysteine biosynthesis such as serine *o*-acetyltransferase (c45775_g1, cysE) and cystathionine gamma-lyase (c72595_g2, CTH) were up-regulated in roots under Co stress (Fig. 4 and Table S9). Taken together, our results showed there were multiple mechanisms involved in detoxification of excess Co in willow, including the inhibition of metal transporter expression and induction of genes related to vacuolar sequestration and metal complexation pathway.

## Methods

### Plant material and morphological parameters measurement

The plant material was collected from an adult male weeping willow (*Salix babylonica*) grown in Beijing Botanical Garden, Institute of Botany, the Chinese Academy of Sciences. Tissue culture of weeping willow materials was performed using the modified protocol as described by Bhojwani^35^. The stem segments with axillary buds (~1.0 cm) were grown in the woody plant medium (WPM) containing 0.2 mg L^−1^ indole-3-butyric acid (IBA) to induce rooting. Tissue cultures were grown at 16/8 h light/dark cycle and 24 °C. Different concentrations of CoCl_2_ (50 μM, 100 μM, 200 μM and 400 μM) were added to WPM for cobalt treatments. WPM without Co was used as control. After 30 days of growth in the Co treatments, the root number and length, stem height and leaf number per seedlings were measured. There were at least 15 replicates for each of Co treatment. The significant differences were analyzed using the *t*-test (one tail, *P* < 0.05). Tissue-cultured seedlings grown under moderate stress conditions of 100 μM Co^2+^ were selected and separated into two parts: roots (root tissues) and tops (stem and leaf tissues), and were used for further physiological and transcriptional analysis.

### Physiological and biochemical characterization

Biomass of roots and tops was measured after drying in an oven at 80 °C for 48 h. Peroxidase activity was assayed by the published method^36,37^. One mL of enzyme extract was mixed with 3 mL of reaction mixture (19 μL of 300 g L^−1^ H_2_O_2_ and 28 μL guaiacol in 50 mL of 100 mmol/L phosphate buffer (pH 6.0), freshly prepared) for 5 min. The absorbance was measured at 470 nm. The soluble sugar content was measured using a phenol-sulfuric acid method^21^. Half an mL of 5% phenol and 2.5 mL of sulfuric acid were added to 0.5 mL of sugar extract and incubated for 10 min at room temperature. Then, the reaction mixture was kept in a boiling water bath for 20 min. After cooling to the room temperature, the absorbance of the extract was measured at 490 nm. The chlorophylls were extracted from leaves using 80% v/v acetone and the concentrations were determined using the method described by Bruuinsma^38^. The absorbance of chlorophyll was measured at 663 and 645 nm. The contents were calculated as: C_chl*a*_ = (12.21 × *A*_663_) − (2.81 × *A*_645_) and C_chl*b*_ = (20.13 × *A*_645_) − (5.03 × *A*_663_). The photosynthetic activity was measured using the Chlorophyll Fluorescence System (MAXI Version; Heinz Walz GmbH, Germany). The gas exchange parameters were measured by the GFS-3000 portable photosynthesis system (Heinz Walz GmbH, Germany) according to the manufacturer’s instructions. The trace element concentrations were determined as described previously^39^ by using Agilent 7700x ICP-MS (Agilent Technologies, Japan) and iCAP 6300 ICP-OES Spectrometer (Thermo Fisher Scientific, USA).

### RNA extraction, cDNA library construction and Illumina transcriptome sequencing

Each seedling was divided into tops (stem and leaf tissues) and roots for RNA extraction. Total RNA samples were extracted using TRNzol Universal Reagent Kit (TIANGEN^®^, China) and subsequently used for mRNA purification and library construction with an NEBNext^®^ Ultra^TM^ RNA Library Prep Kit for Illumina (NEB, USA) following the manufacturer’s instructions. The samples were sequenced on an Illumina Hiseq™ 4000 by the Beijing Allwegene Technology Company Limited (Beijing, China). Each sample yielded more than 6 GB of data. Each experiment had three biological replicates for RNA-seq and qRT-PCR.

### Transcriptome sequencing data analysis

The quality control of raw reads was using an NGS QC Toolkit v2.3.3^40^ software and the in-house scripts to remove the reads containing adaptor sequences, non-ATGC bases greater than 10% or Q20 bases less than 50%. The high-quality clean reads data were assembled by Trinity v2.3.2^41^ with default parameters and then evaluated by BUSCO v3.0.2^42^. The gene expression level was calculated by RSEM v1.3.0^43^ and the differential expression analysis was performed using the edgeR v3.18.1^44^ package. Read counts values were assigned to each unigene to normalize expression^45^. We defined differential expressed gene (DEG) as a gene with 2-fold expression changes between the treatment and control samples and false discovery rate (FDR) of less than 0.05.

The gene function annotation was performed using BLAST v2.2.28+^46^ by aligning all unigene sequences to NCBI (National Center for Biotechnology Information) NR (ftp://ftp.ncbi.nlm.nih.gov/blast/db), NCBI NT (ftp://ftp.ncbi.nlm.nih.gov/blast/db), UniProtKB Swiss-Prot (http://www.uniprot.org/downloads) and KOG (ftp://ftp.ncbi.nih.gov/pub/COG/KOG) databases. The Pfam annotations were performed using the HMMER 3.1b2 (http://hmmer.org). All the expected thresholds were 10^−5^. KEGG annotations were done using the BBH (bi-directional best hit) method on the KEGG Automatic Annotation Server mingVer. 2.1^47^. Gene Ontology (GO) annotations were performed by Blast2GO v2.5^48^. Transcription factor prediction and annotation were performed using the Plant Transcription Factor Database v4.0^49^. The GO enrichment analysis was performed using the topGO v2.28.0^50^ package and the KEGG enrichment analysis was performed using KOBAS v3.0.3^51^. All software parameters were used with the default settings.

### qRT-PCR verification

To verify the accuracy of the RNA-seq results, twelve DEGs were selected randomly for qRT-PCR. The β-actin gene was used as internal reference gene. Primers are listed in Table S13. PCR amplification used TaKaRa SYBR^®^ Premix Ex Taq™ II (Tli RNaseH Plus) and was performed on the ABI Step One Plus Real-Time PCR. The relative expression level was calculated using the 2^−ΔΔ*C*^_T_ method^52^. The experiment comprised three independent biological replicates and three technical replicates.

## Supplementary information

Supplementary information

Supplementary information2 

Supplementary information3

Supplementary information4

Supplementary information5 

Supplementaryinformation6

Supplementary information7.

Supplementary information8

Supplementary .information9

Supplementary information10

Supplementaryinformation11

Supplementary information12

Supplementary information13

Supplementary information14

## Data Availability

The raw sequence data presented in this study have been submitted to the NCBI Sequence Read Archive (http://trace.ncbi.nlm.nih.gov/Traces/sra/sra.cgi?view=studies), and the accession numbers are SRR6793894 to SRR6793905. The assembled transcript sequences (unigenes) and gene function annotation information can be obtained from the GitHub hosting platform (https://github.com/Wang-Yi-Ming/CobaltStress-RNA-seq).
