# Supplementary material for: Physiological and transcriptomic analysis provide novel insight into cobalt stress responses in willow
Source: Sci Rep. 2020 Feb 11;10:2308. doi: 10.1038/s41598-020-59177-y (PMC7012891; doi:10.1038/s41598-020-59177-y)
Supplement: Supplementary file 1 — Supplementary information [file 41598_2020_59177_MOESM1_ESM.pdf]

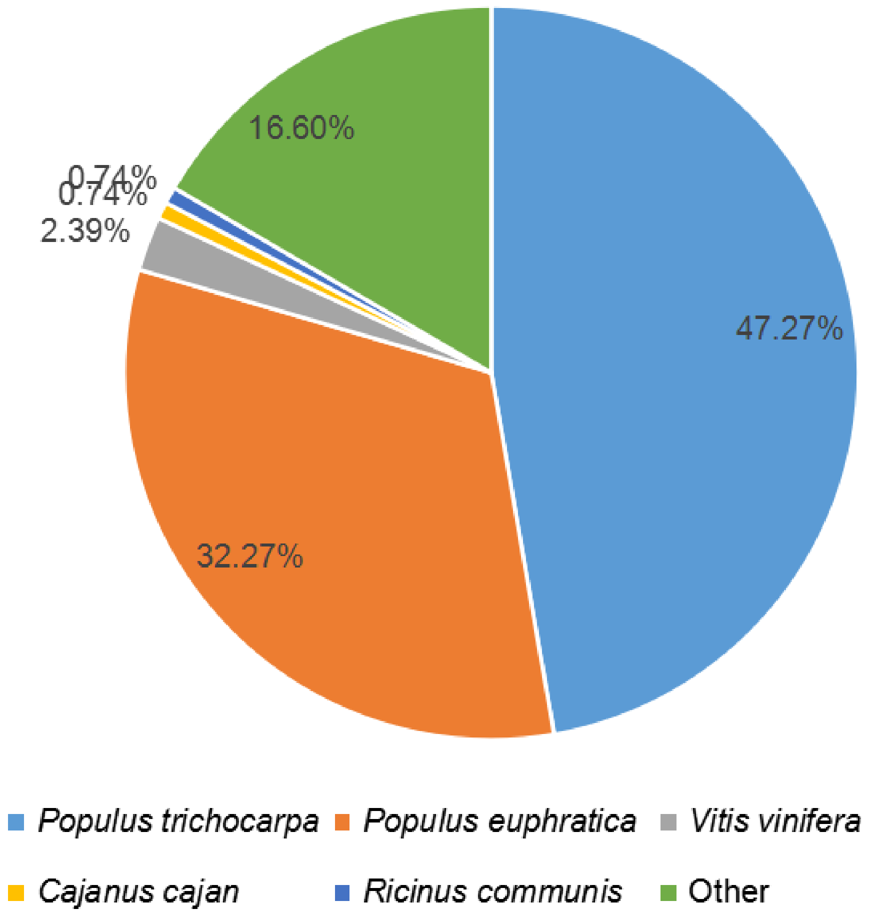

Figure S1. The distribution of the top BLAST hits of unigenes from different species. Distribution are shown as the percentage of the total homologous sequences (with an E-value ≤ 1.0 e^{-5}). We used all nucleotide sequences in the NCBI NT database for homology search and extracted the best hit of each unigene for analysis.

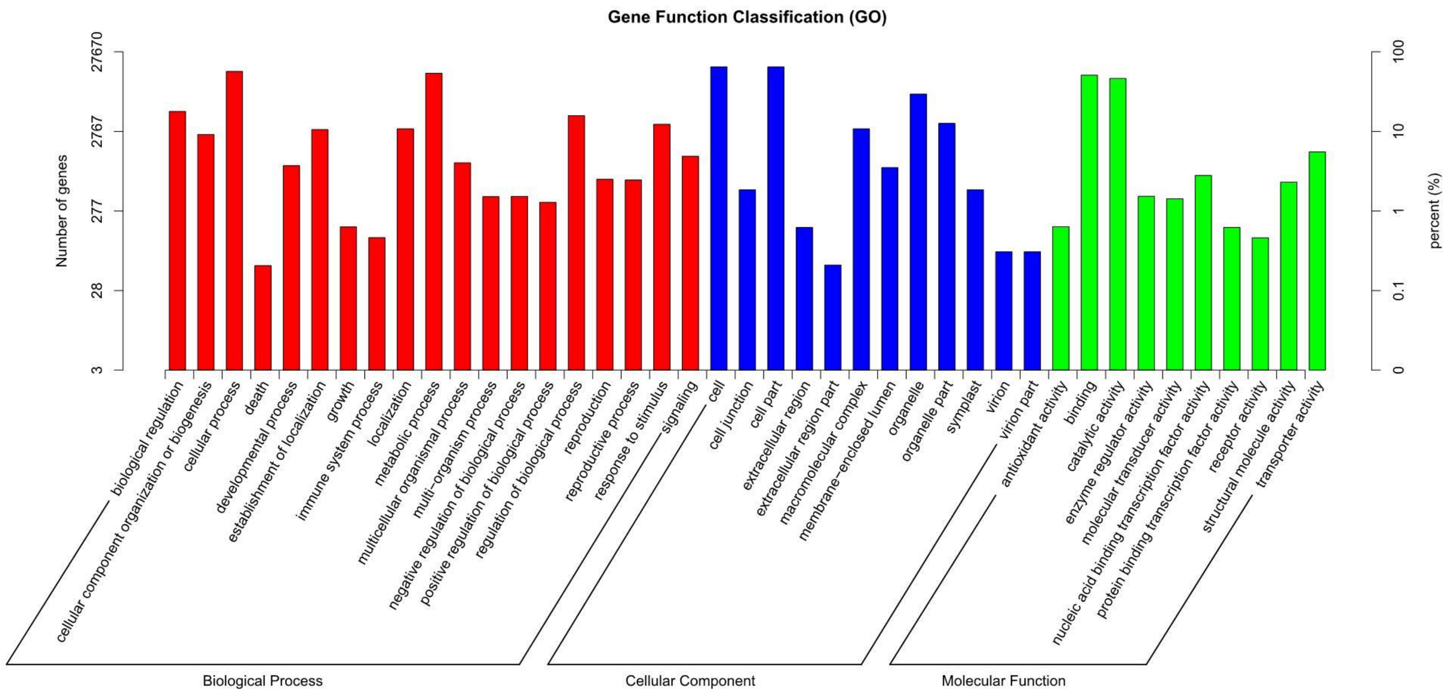

Figure S2. Histogram of Gene Ontology (GO) classification for unigenes identified from transcriptomic analysis. The results are summarized in three main categories: Biological Process (red), Cellular Component (blue) and Molecular Function (green). Percentage on the right y-axis indicates the proportion of unigenes that have GO annotations.

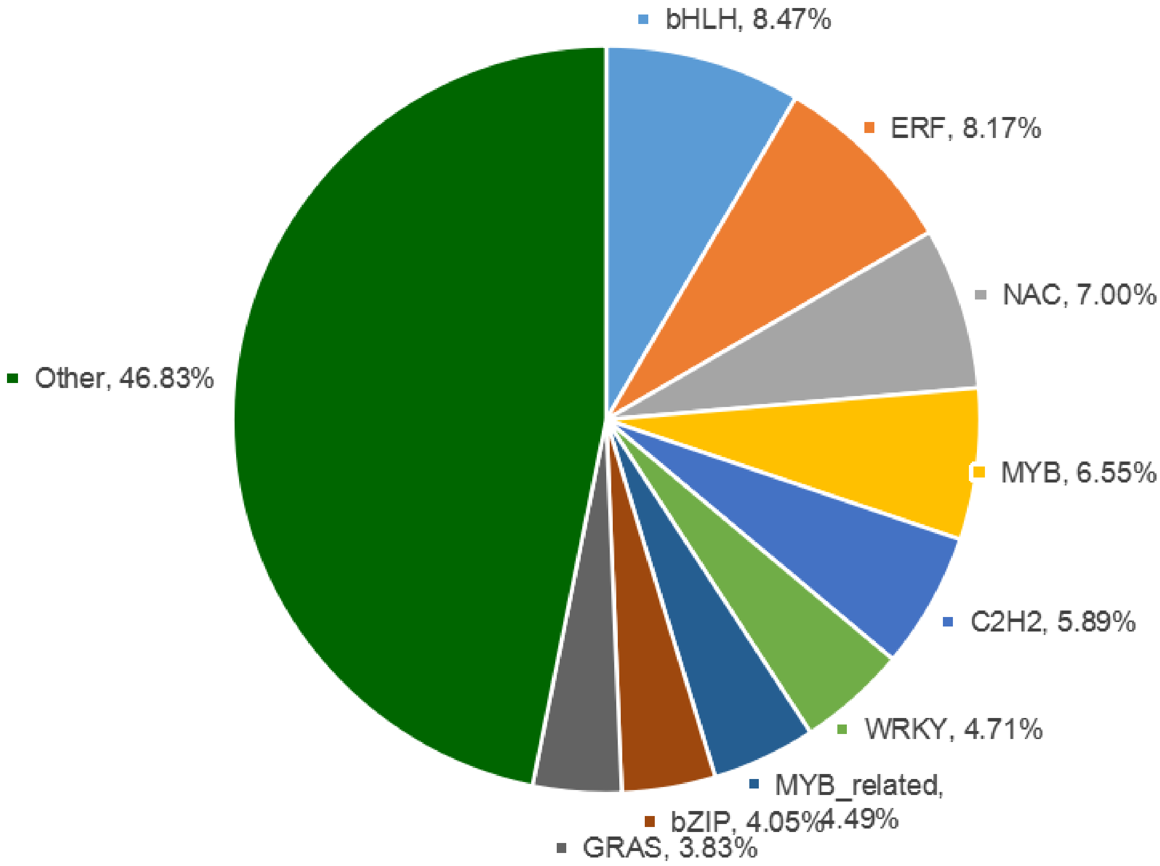

Figure S3. The abundance of different transcription factor families in willow. Percentage is calculated as the TF number of each TF family to the total number of identified transcription factors. Only the top nine abundant TF families are shown.

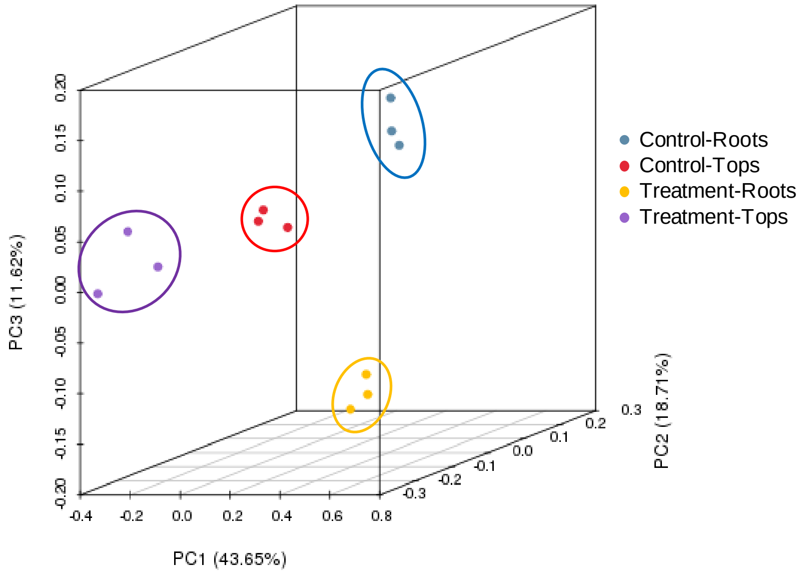

Figure S4. Principal components analysis of transcriptomic data. Principal components analysis (PCA) of reads count values from RNA-seq. Each dot represents one sample set and three biological replicates are enclosed with a circle.

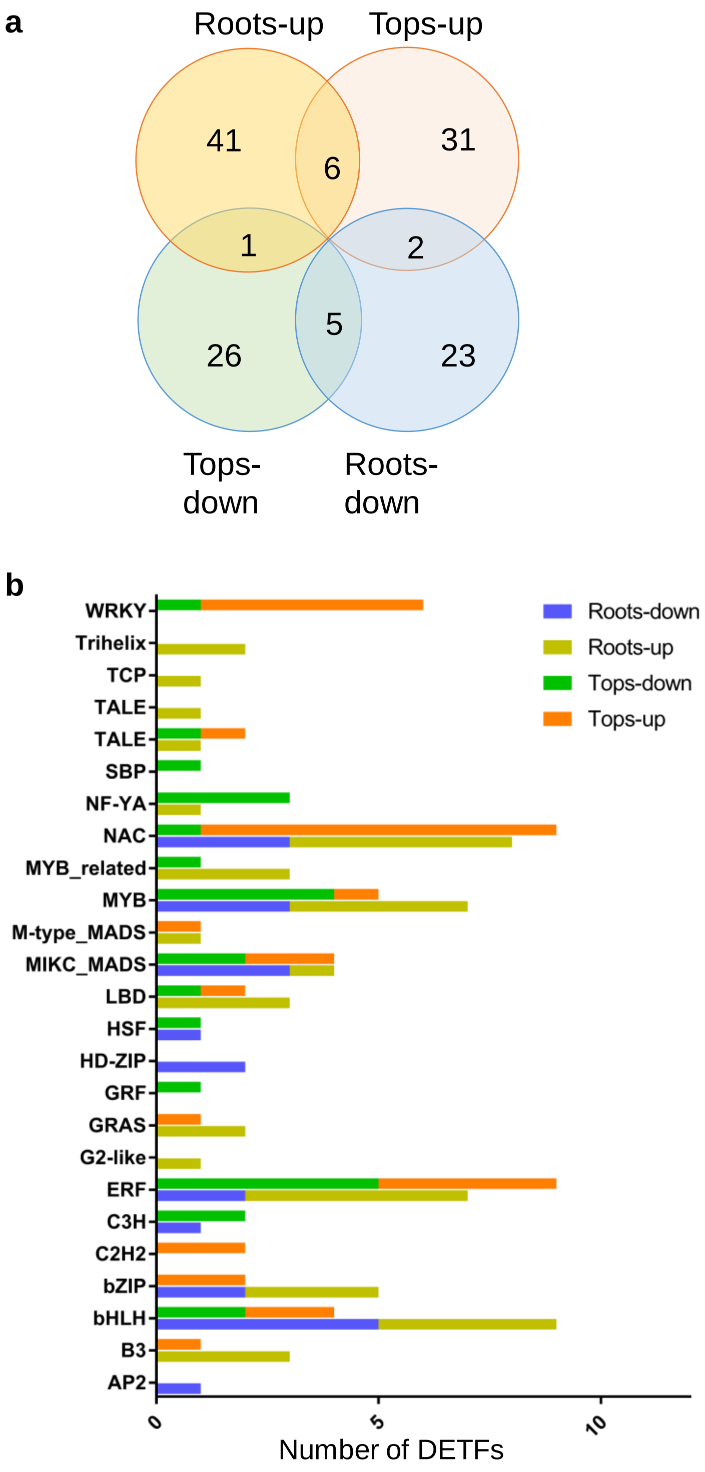

Figure S5. Summary of identified differentially expressed transcription factors (DETFs) in roots and tops in willow in responding to Co stress. (**a**) Venn diagram shows the numbers of specific and common DETFs in roots and tops with Co treatment. (**b**) The distribution of DETFs of various TF families in responding to Co stress.

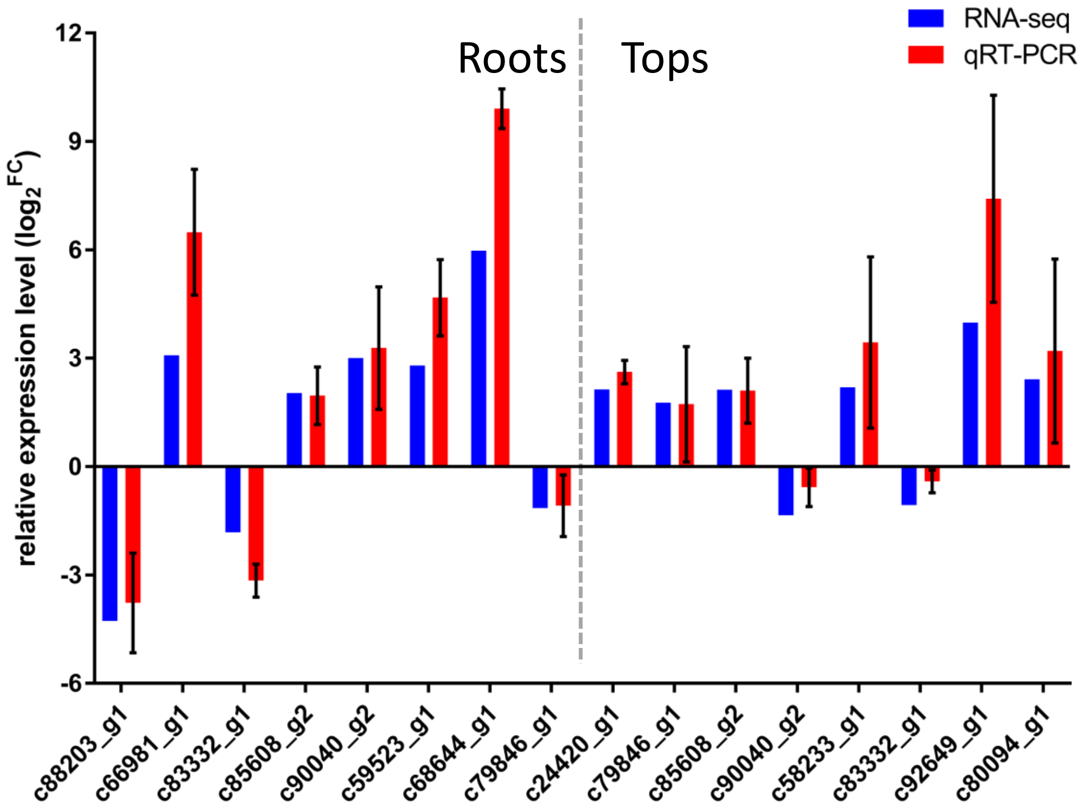

Figure S6. qRT-PCR verification of twelve selected DEGs in willow treated with Co stress. On the left and right side of the dash line are expression profiles of DEGs in roots and tops (leaf and stem tissues), respectively. Within the twelve genes, four of them were differentially expressed in both roots and Tops.

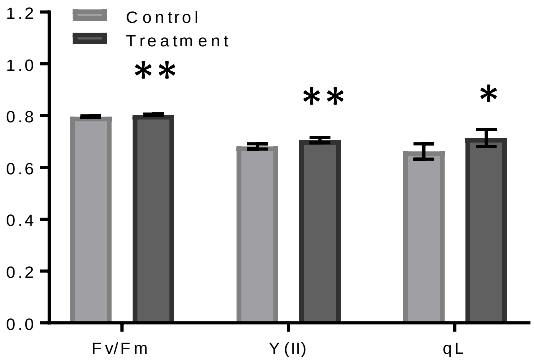

Figure S7. Effects of cobalt stress on the photosynthesis in willow. F_v/F_m, maximum quantum efficiency of photosystem II photochemistry; Y(II), operating efficiency of photosystem II; qL, photochemical quenching coefficient. Data represent mean ± SD (n=3). Asterisks indicate the significant difference between treatment and control (*, P≤0.05; **, P≤0.01, t-test).
